# PPARβ/δ Augments IL-1β-Induced COX-2 Expression and PGE2 Biosynthesis in Human Mesangial Cells via the Activation of SIRT1

**DOI:** 10.3390/metabo12070595

**Published:** 2022-06-27

**Authors:** Yaqing Li, Rong Cao, Tingting Gu, Cong Cao, Tingyue Chen, Youfei Guan, Xiaoyan Zhang

**Affiliations:** 1 Department of Physiology and Pathophysiology, Advanced Institute of Medical Sciences (AIMS), School of Basic Medical Sciences, Dalian Medical University, Dalian 116044, China; liyaqing1114@163.com (Y.L.); caocong10140826@163.com (C.C.); ctydyx2022@163.com (T.C.); 2 Department of Nephrology, The First Affiliated Hospital of Shenzhen University, The Second People’s Hospital of Shenzhen, Shenzhen 518039, China; caorong19870118@163.com; 3 Division of Nephrology, Nantong University Affiliated Hospital, Nantong 226001, China; cuteg89@163.com; 4 Health Science Center, East China Normal University, Shanghai 200241, China

**Keywords:** nuclear receptor, cyclooxygenase, sirtuin 1, inflammation, glomerular mesangial cell

## Abstract

Peroxisome proliferator-activated receptor β/δ (PPARβ/δ), a ligand-activated nuclear receptor, regulates lipid and glucose metabolism and inflammation. PPARβ/δ can exert an anti-inflammatory effect by suppressing proinflammatory cytokine production. Cyclooxygenase-2 (COX-2)-triggered inflammation plays a crucial role in the development of many inflammatory diseases, including glomerulonephritis. However, the effect of PPARβ/δ on the expression of COX-2 in the kidney has not been fully elucidated. The present study showed that PPARβ/δ was functionally expressed in human mesangial cells (hMCs), where its expression was increased by interleukin-1β (IL-1β) treatment concomitant with enhanced COX-2 expression and prostaglandin E2 (PGE2) biosynthesis. The treatment of hMCs with GW0742, a selective agonist of PPARβ/δ, or the overexpression of PPARβ/δ via an adenovirus-mediated approach significantly increased COX-2 expression and PGE2 production. PPARβ/δ could further augment the IL-1β-induced COX-2 expression and PGE2 production in hMCs. Moreover, both PPARβ/δ activation and overexpression markedly increased sirtuin 1 (SIRT1) expression. The inhibition or knockdown of SIRT1 significantly attenuated the effects of PPARβ/δ on the IL-1β-induced expression of COX-2 and PGE2 biosynthesis. Taken together, PPARβ/δ could augment the IL-1β-induced COX-2 expression and PGE2 production in hMCs via the SIRT1 pathway. Given the critical role of COX-2 in glomerulonephritis, PPARβ/δ may represent a novel target for the treatment of renal inflammatory diseases.

## 1. Introduction

PPARβ/δ is a ligand-activated transcription factor that belongs to the nuclear hormone receptor family [1,2,3]. PPARβ/δ is ubiquitously expressed in almost all examined tissues. Previous studies have demonstrated that PPARβ/δ plays important roles in the regulation of cell proliferation and differentiation, lipid and glucose metabolism, fatty acid oxidation, female reproduction, and cancer [4,5,6,7,8,9]. Recently, it has been reported that PPARβ/δ takes part in the progression of inflammatory diseases [10,11,12], such as inflammatory bowel disease [12,13,14], skin inflammation [15,16], and many other tissues’ inflammation [4,8,11,17,18,19,20]. In particular, increasing evidence has suggested that PPARβ/δ also plays an important role in renal inflammatory diseases. The activation of PPARβ/δ delayed the progression of streptozotocin (STZ)-induced diabetic nephropathy through the inhibition of inflammatory processes [21]. The PPARβ/δ agonist GW0742 reduced macrophage infiltration in the glomerulus and decreased the expression of monocyte chemotactic protein 1 (MCP-1), transforming growth factor-β (TGF-β), and osteopontin [22]. PPARβ/δ was also found to attenuate renal dysfunction, leukocyte infiltration, and the expressions of IL-6 and TNF-α in a model of renal ischemia-reperfusion (I/R) in STZ-induced diabetic rats. The beneficial effect of GW0742 was found to be attenuated by the selective PPARβ/δ antagonist GSK0660 [23].

Glomerulonephritis represents the leading cause of kidney failure among east Asian populations [24,25]. It is generally believed that glomerular mesangial cells (MCs) play an important role in the pathogenesis of glomerulonephritis [26,27]. As specialized, smooth muscle cells, mesangial cells support the capillaries of the renal glomerulus and regulate the glomerular filtration rate. Moreover, MCs have many other functions, including the generation of prostaglandins (PGs), which serve as major mediators of inflammation. Cyclooxygenases (COXs) are key enzymes in the conversion of arachidonic acid to PGs and other eicosanoids. Thus far, two COX subtypes have been identified, i.e., a constitutive one named COX-1 and an inducible one called COX-2. Classically, COX-2 is induced in inflammatory conditions with significant increase in the production of cyclooxygenase-derived prostaglandin E2, a well-known dominant proinflammatory lipid mediator [16,28,29]. In a rat model of mesangioproliferative glomerulonephritis, selective COX-2 inhibition by either rofecoxib or celecoxib led to more profound mesangiolysis and albuminuria [30]. Moreover, several studies have reported that, in cultured MCs of rodents and humans, COX-2 expression and prostaglandin synthesis could be stimulated by IL-1β, a critical proinflammatory cytokine that plays an important role in glomerular inflammation and injury [31,32,33,34].

Both in vitro and in vivo studies have clearly shown that the nuclear receptor PPARβ/δ plays an important role in the progression of inflammatory diseases, and the activation of PPARβ/δ can upregulate the expression of COX-2 in tumor cells [35,36]. To date, the role of PPARβ/δ in glomerular inflammation is not clear. Whether PPARβ/δ is involved in glomerular COX-2 expression and PGE2 production in mesangial cells also remain largely unknown. The current study aims to clarify the potential role and the underlying mechanism of PPARβ/δ in IL-1β-induced COX-2 expression and PGE2 biosynthesis in cultured human mesangial cells.

## 2. Results

### 2.1. PPARβ/δ Is Functionally Expressed in hMCs

**Figure 1:**
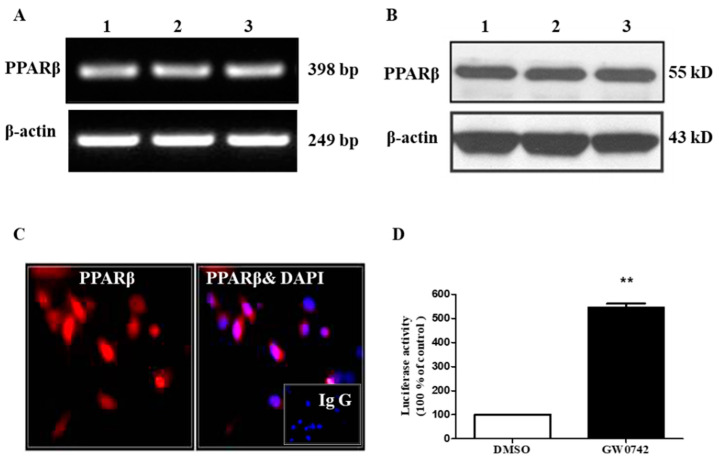
PPARβ/δ is functionally expressed in human mesangial cells. (**A**,**B**) Constitutive expression of PPARβ/δ in mesangial cells. Real-time PCR and Western blot assays were used to measure PPARβ/δ mRNA (**A**) and protein (**B**) expression in 3 preparations of mesangial cells. (**C**) The nuclear localization of PPARβ/δ in human mesangial cells as assessed by immunofluorescence. Red color represents PPARβ/δ immunoreactivity. The nuclei were stained with DAPI in blue. IgG was used as a negative control. (**D**) The effect of the PPARβ/δ agonist GW0742 on PPRE-luciferase activity. The cells were cotransfected with PPRE-luciferase plasmid and β-galactosidase vector for 24 h, followed by treatment with GW0742 (1 μM) for 6 h. The luciferase activity was measured as described in the experimental procedure. ** *p* < 0.01 compared to DMSO; *n* = 3. PPARβ: PPARβ/δ.

The expression of PPARβ/δ mRNA and protein was evident as assessed by qRT-PCR and Western blot assays (Figure 1A,B). An immunofluorescence study further demonstrated that PPARβ/δ protein was mainly localized in the nuclei of hMCs, with little expression in the cytoplasm (Figure 1C). A PPRE-luciferase reporter assay further indicated that the PPARβ/δ-specific agonist GW0742 significantly increased the activity of the PPRE-driven luciferase reporter by ~five-fold, suggesting that the hMCs had functional PPARβ/δ transcriptional activity (Figure 1D). Collectively, these results revealed that PPARβ/δ was constitutively expressed and functional in hMCs.

### 2.2. PPARβ/δ Increases COX-2 Expression and PGE2 Production in hMCs

**Figure 2:**
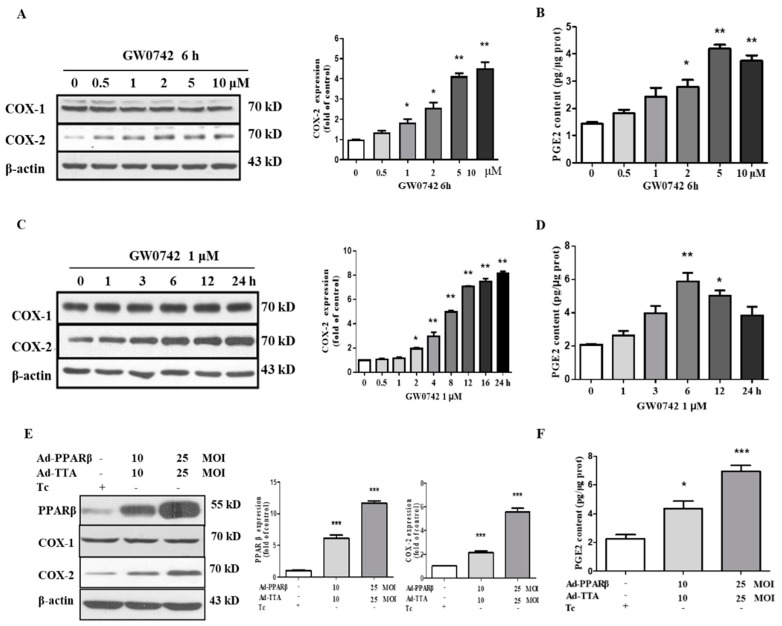
PPARβ/δ increases COX-2 expression and PGE2 production in human glomerular mesangial cells. (**A**) PPARβ/δ activation dose-dependently induced COX-2 expression. The cells were treated with various concentrations of GW0742 (0.5, 1, 2, 5, and 10 μM) for 6 h. COX-1 and COX-2 protein expressions were examined using Western blot. * *p* < 0.05 and ** *p* < 0.01 compared to the control (0 ng/mL). (**B**) GW0742 dose-dependently induced PGE2 production. * *p* < 0.05 and ** *p* < 0.01 compared to the untreated control; *n* = 4. (**C**) A Western blot assay demonstrated that GW0742 (1 μM) time-dependently induced COX-2 expression. * *p* < 0.05 and ** *p* < 0.01 compared to the control (0 h). (**D**) GW0742 treatment increased PGE2 production in a time-dependent manner. * *p* < 0.05 and ** *p* < 0.01 vs. the untreated control; *n* = 4. (**E**) PPARβ/δ overexpression increased COX-2 expression as assessed by Western blot. The cells were infected with Ad-PPARβ/δ and Ad-TTA with or without 0.1 μg/mL tetracycline for 24 h. *** *p* < 0.001 compared to the control. (**F**) The adenovirus-mediated overexpression of PPARβ/δ induced PGE2 production. * *p* < 0.05, and *** *p* < 0.001 compared to the control; *n* = 4. Ad-TTA: adenovirus-tetracycline-controlled transcriptional activator; Tc: tetracycline.

Previous studies have demonstrated that PPARβ/δ activation upregulates COX-2 expression in tumor cells [37,38]. However, the effect of PPARβ/δ on COX-2 expression in other cell types remains unknown. In the present study, we found that the treatment of hMCs with GW0742, a specific PPARβ/δ agonist, dose-dependently upregulated COX-2 protein expression with little effect on COX-1 expression (Figure 2A). Consistent with the increased COX-2 expression, the PGE2 production was also significantly increased by the GW0742 treatment (Figure 2B). Meanwhile, GW0742 increased the COX-2 expression and PGE2 production in a time-dependent manner (Figure 2C,D). To further confirm the effect of PPARβ/δ on COX-2 expression, PPARβ/δ was overexpressed in the hMCs via an adenovirus-based approach. The results showed that PPARβ/δ overexpression also significantly increased the COX-2 protein levels and PGE2 release (Figure 2E,F). Together, these results strongly indicated that PPARβ/δ can increase the expression of COX-2 and the production of PGE2 in human mesangial cells.

### 2.3. IL-1β Induces PPARβ/δ Expression in hMCs

**Figure 3:**
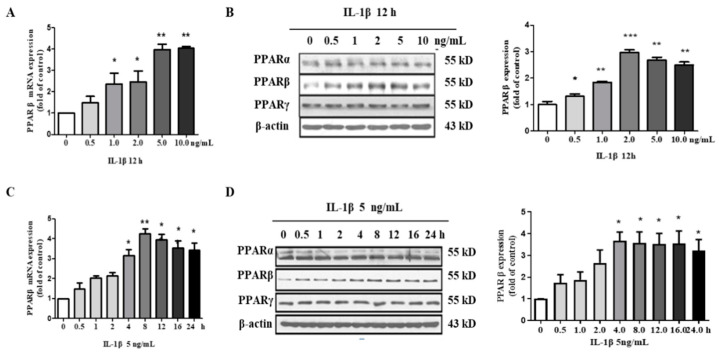
IL-1β induces PPARβ/δ expression in human mesangial cells. (**A**,**B**) Real-time PCR and Western blot assays demonstrated that IL-1β induced PPARβ/δ mRNA (**A**) and protein (**B**) expressions in a dose-dependent manner. The cells were treated with various concentrations of IL-1β (0.5, 1, 2, 5, and 10 ng/mL) for 12 h. The protein expressions of PPARα, PPARβ/δ, and PPARγ were examined using Western blot. (**C**,**D**) Real-time PCR and Western blot assays demonstrated that IL-1β induced PPARβ/δ mRNA (**C**) and protein (**D**) expressions in a time-dependent manner. The cells were incubated with IL-1β (5 ng/mL) for the indicated time periods. * *p* < 0.05, ** *p* < 0.01, and *** *p* < 0.001 compared to the control (0 ng/mL or 0 h); *n* = 4. PPARβ: PPARβ/δ.

Given the important role of PPARβ/δ in the regulation of inflammation in many cell types, it is reasonable to hypothesize that PPARβ/δ may also be involved in IL-1β-induced inflammatory process in hMCs. To explore the effect of IL-1β on PPARβ/δ expression, the hMCs were stimulated with various concentrations of IL-1β for 12 h. The results showed that IL-1β significantly increased both the mRNA and protein expressions of PPARβ/δ in a dose-dependent manner (Figure 3A,B). Unlike PPARβ/δ, the PPARα and PPARγ protein levels remained unaffected by the IL-1β treatment (Figure 3B). Moreover, IL-1β (5 ng/mL) significantly upregulated the PPARβ/δ expression in a time-dependent manner (Figure 3C,D). These findings demonstrated that IL-1β can induce PPARβ/δ expression in hMCs.

### 2.4. PPARβ/δ Activation Augments IL-1β-Induced COX-2 Expression and PGE2 Production in hMCs

**Figure 4:**
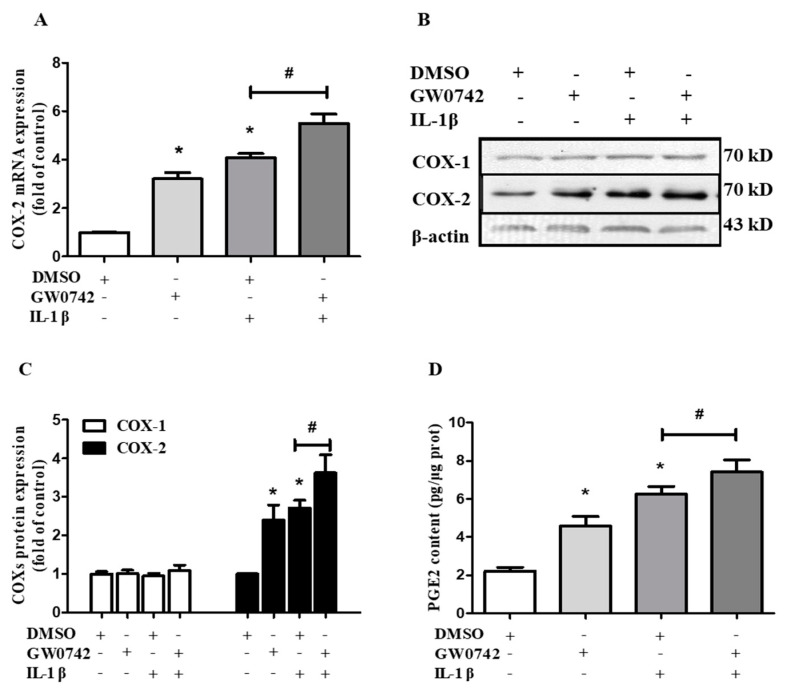
PPARβ/δ augments IL-1β-induced COX-2 expression and PGE2 production. The cells were incubated with DMSO or GW0742 (1 μM) for 6 h and then stimulated with or without IL-1β (5 ng/mL) for 12 h. (**A**) A real-time PCR analysis showed the induction of COX-2 mRNA expression by the PPARβ/δ agonist GW0742. (**B**,**C**) A Western blot assay demonstrated that GW0742 induced COX-2 protein expression with little effect on the COX-1 levels. (**D**) GW0742 treatment significantly augmented IL-1β-induced PGE2 production. * *p* < 0.05 vs. DMSO; # *p* < 0.05 vs. IL-1β; *n* = 4.

To further explore the role of PPARβ/δ in IL-1β-induced COX-2 expression and PGE2 production, human mesangial cells were pretreated with or without GW0742 (1 μM) for 6 h, followed by stimulation with IL-1β (5 ng/mL) for 12 h. The pretreatment of the cells with GW0742 significantly augmented the IL-1β-induced COX-2 mRNA expression compared with GW0742 or IL-1β treatment alone (Figure 4A). Although GW0742 or IL-1β treatment alone could increase the COX-2 protein levels, the pretreatment with GW0742 further augmented the IL-1β-induced COX-2 protein expression (Figure 4B,C). Consistently, GW0742 also enhanced the IL-1β-induced PGE2 production in the media of the cultured cells (Figure 4D). Together, these results indicate that PPARβ/δ activation increased the COX-2 expression and activity both under the basal condition and following the IL-1β treatment.

### 2.5. PPARβ/δ Increases the Expression of SIRT1 in hMCs

**Figure 5:**
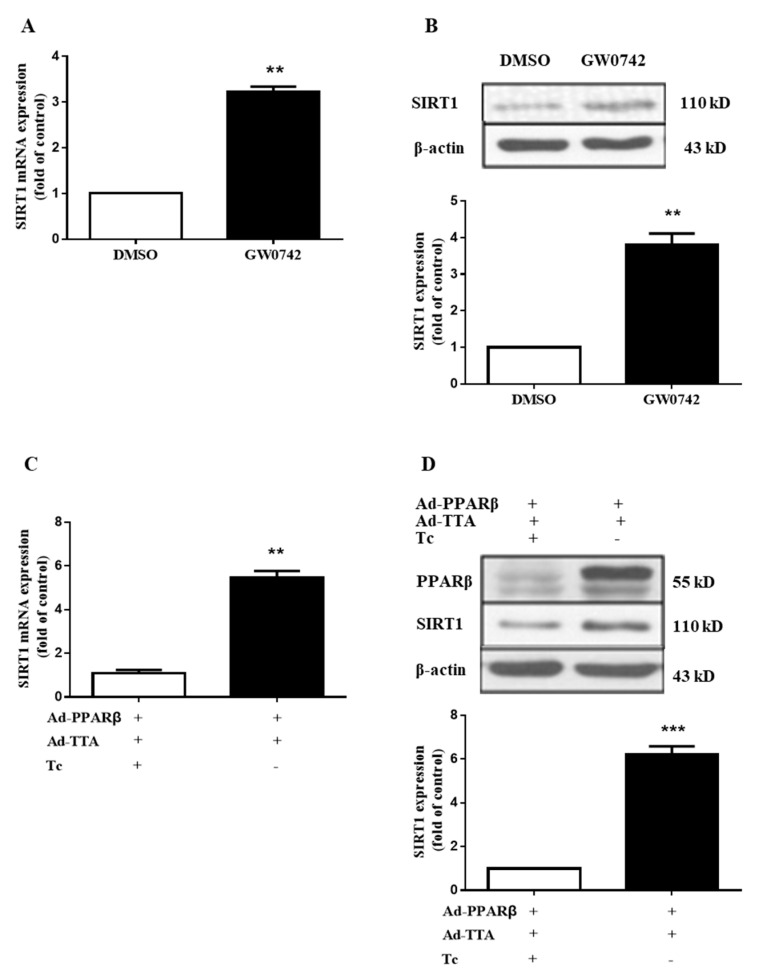
PPARβ/δ increases expression of SIRT1 in glomerular mesangial cells. (**A**,**B**) PPARβ/δ activation by GW0742 increased SIRT1 mRNA (**A**) and protein (**B**) expressions. The cells were treated with or without GW0742 (1 μM) for 6 h. SIRT1, COX-1, and COX-2 expression levels were analyzed with real-time PCR and Western blot. ** *p* < 0.01 vs. DMSO; *n* = 3. (**C**,**D**) Adenovirus-mediated PPARβ/δ overexpression upregulated SIRT1 mRNA (**C**) and protein (**D**) expressions. The cells were infected with Ad-PPARβ/δ and Ad-TTA (25 MOI) with or without 0.1 μg/mL tetracycline for 24 h. PPARβ/δ, SIRT1, COX-1, and COX-2 were analyzed with real-time PCR and Western blot. *** *p* < 0.001 vs. Ad-PPAR β+ Ad-TTA + Tc; *n* = 3. PPARβ: PPARβ/δ.

It was reported that SIRT1 activation increased renal medullary interstitial cell COX-2 expression both in vitro and in vivo, while SIRT1 deficiency-attenuated oxidative stress-induced COX-2 expression in cultured mouse renal medullary interstitial cells [37]. In addition, PPARβ/δ was found to increase SIRT1 gene transcription via a canonical Sp1 binding site [39]. Based on these findings, we hypothesized that a PPARβ/δ-SIRT1-COX-2 axis may exist in human mesangial cells. In fact, we observed a stimulatory effect of PPARβ/δ on SIRT1 expression in hMCs. Following the treatment of hMCs with 1 μM GW0742 for 6 h, the mRNA and protein expressions of SIRT1 were significantly increased (Figure 5A,B). Similarly, adenovirus-mediated PPARβ/δ overexpression also markedly increased the SIRT1 expression at both the mRNA and protein levels (Figure 5C,D). These results strongly indicated that PPARβ/δ can elevate SIRT1 mRNA and protein expressions in human mesangial cells.

### 2.6. SIRT1 Inhibition Blocks the Stimulatory Effect of PPARβ/δ on IL-1β-Induced COX-2 Expression and PGE2 Production in hMCs

**Figure 6:**
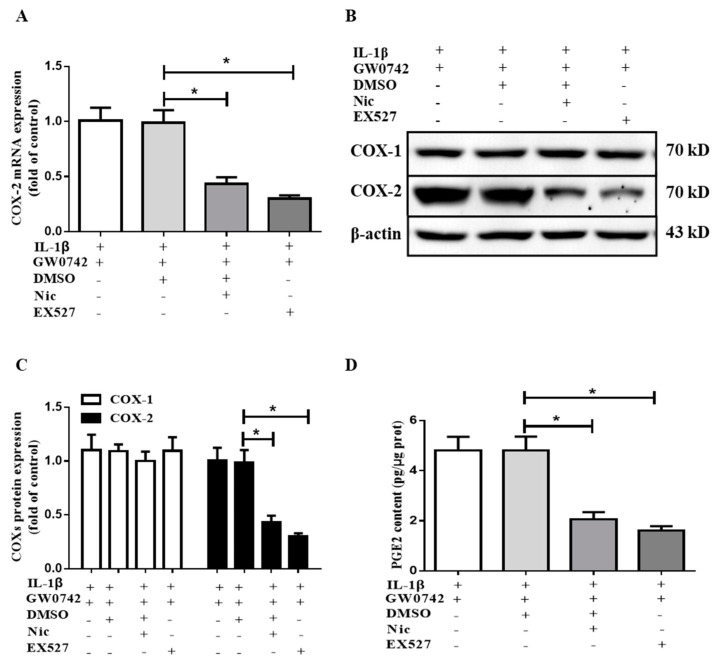
SIRT1 inhibition blocks the stimulatory effect of PPARβ/δ on IL-1β-induced COX-2 expression and PGE2 production in human mesangial cells. The cells were pretreated with GW0742 (1 μM) in the presence or absence of the SIRT1 inhibitors NIC (niconinamide, 5 mM) or EX527 (1 μM) for 6 h, followed by treatment with IL-1β (5 ng/mL) for 12 h. (**A**) The SIRT1 inhibition significantly attenuated the stimulatory effect of PPARβ/δ on the IL-1β-induced COX-2 expression. The expression of COX-2 mRNA was analyzed with real-time PCR. (**B**,**C**) A Western blot assay showed that SIRT1 inhibition blocked the stimulatory effect of PPARβ/δ on the IL-1β-induced COX-2 protein expression with little effect on COX-1. (**D**) The effect of SIRT1 inhibition on PGE2 production. * *p* < 0.05 vs. IL-1β+ GW0742 + DMSO; *n* = 4.

To investigate the role of SIRT1 in the effect of PPARβ/δ on the IL-1β-induced upregulation of COX-2, two SIRT1 inhibitors, including NIC (niconinamide) and EX527, were used. The cells were pretreated with GW0742 (1 μM) in the presence or absence of the SIRT1 inhibitor NIC (niconinamide, 5 mM) or EX527 (1 μM) for 6 h, followed by treatment with IL-1β (5 ng/mL) for 12 h. We found that pretreatment with either NIC or EX527 decreased the COX-2 mRNA expression induced by IL-1β and GW0742 (Figure 6A). Compared with the IL-1β + GW0742 group, the protein abundance of COX-2 was decreased to 43% and 30% in the IL-1β + GW0742 + NIC group and the IL-1β + GW0742+ EX527 group, respectively (Figure 6B,C). Consistently, PGE2 production in the media of the NIC and EX527 treatment groups was significantly reduced compared with the group without treatment with the SIRT1 inhibitors (Figure 6D). Together, these findings demonstrated that the augmentation of PPARβ/δ on IL-1β-induced COX-2 expression and PGE2 production is mediated by SIRT1.

### 2.7. SIRT1 Knockdown Blocks the Effect of PPARβ/δ on IL-1β-Induced COX2 Expression and PGE2 Production in hMCs

**Figure 7:**
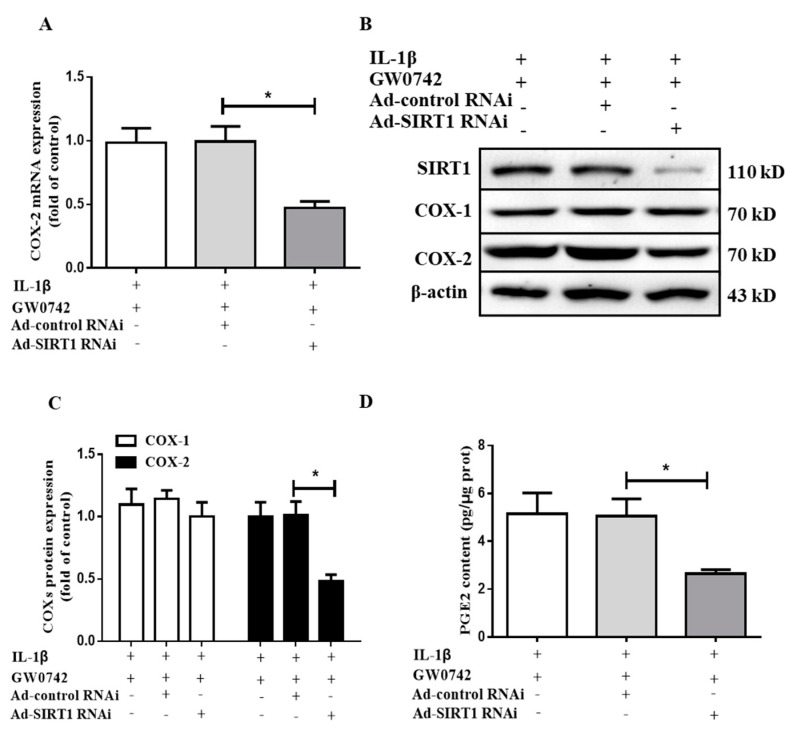
SIRT1 gene silencing abolishes the stimulatory effect of PPARβ/δ on IL-1β-induced COX-2 expression and PGE2 production in human mesangial cells. After infection with Ad-siSIRT1 (10 MOI) or Ad-siControl (10 MOI), the cells were pretreated with GW0742 (1 μM) for 6 h and then treated with IL-1β (5 ng/mL) for 12 h. (**A**) Ad-siRNA-mediated SIRT1 gene knockdown abolished the stimulatory effect of PPARβ/δ on IL-1β-induced COX-2 mRNA expression, as assessed with real-time PCR. (**B**,**C**) The effect of SIRT1 gene knockdown on SIRT1, COX-1, and COX-2 protein expressions was measured using Western blot. (**D**) The effect of SIRT1 gene silencing on PGE2 production. * *p* < 0.05 vs. IL-1β + GW0742 + Ad-siControl; *n* = 4.

To further support the finding that SIRT1 mediated the stimulatory effect of PPARβ/δ on the IL-1β-induced COX-2 expression, the cells infected with Ad-siSIRT1 (10 MOI) or Ad-siControl (10 MOI) were pretreated with GW0742 (1 μM) for 6 h and then treated with IL-1β (5 ng/mL) for 12 h. As expected, the Ad-siSIRT1 treatment markedly decreased the SIRT1 protein expression. Similar to the findings on SIRT1 inhibition, the knockdown of the SIRT1 expression significantly attenuated the GW0742- and IL-1β-induced COX-2 expression at both the mRNA (Figure 7A) and protein (Figure 7B,C) levels. The effects of GW4064 and IL-1β on PGE2 production were also markedly reduced (Figure 7D). Together, these results further demonstrated that PPARβ/δ enhances the IL-1β-induced COX-2 expression and PGE2 production in hMCs via a SIRT1-dependent pathway.

## 3. Discussion

PPARβ/δ, a ligand-activated nuclear receptor, plays a critical role in regulating lipid and glucose metabolism. Growing evidence also has indicated that PPARβ/δ can exert an anti-inflammatory effect by suppressing proinflammatory cytokine production [38,40]. However, whether PPARβ/δ also suppresses the inflammatory process in glomerulonephritis remains unclear. The present study demonstrated that PPARβ/δ was constitutively expressed in human glomerular mesangial cells, where it promoted COX-2 expression and PGE2 production both under the basal condition and after IL-1β treatment. We further revealed that the stimulatory effect of PPARβ/δ on the IL-1β-induced COX-2 expression was mediated by the activation of SIRT1, a highly conserved NAD(+)-dependent deacetylase. Collectively, our findings indicated that PPARβ/δ may enhance the IL-1β-mediated activation of COX-2 to promote PGE2 production in glomerular mesangial cells.

COX-2-derived PGE2 is a potent proinflammatory lipid mediator that is linked to numerous pathophysiological conditions, including cancer, cardiovascular disease, atherosclerosis, and glomerulonephritis [36,39,41]. It has been well-documented that COX-2 expression can be upregulated by various cytokines, including IL-1β in ganglia cells [42] and glomerular mesangial cells [39,41]. As a proinflammatory cytokine, IL-1β plays a critical role in the pathogenesis of glomerulonephritis, including immune complex glomerulonephritis, nephrotoxic serum nephritis, IgA nephritis, progressive crescentic glomerulonephritis, and lupus nephritis [43,44,45,46,47,48]. The present study confirmed the previous finding that IL-1β induces COX-2 expression and PGE2 production, suggesting that the COX-2–PGE2 pathway may mediate the pathological effect of IL-1β in human glomerular mesangial cells and in multiple types of glomerulonephritis.

As a nuclear receptor transcriptional factor, PPARβ/δ is involved in many physiological and pathological processes [1,2,3]. Increasing evidence has suggested that PPARβ/δ plays an important role in the regulation of inflammatory responses [10,11,12]. PPARβ/δ agonists have been shown to induce the inducible nitric oxide synthase (iNOS) expression in mesangial cells [49] and to upregulate the expression of COX-2 in colon cancer cells [9,50]. Consistent with our finding, Kim et al. previously reported that interleukin 1 (IL-1) increased PPARβ/δ levels in Hep3B human hepatoma cells [51]. However, they found a significant decrease in the expressions of PPARα and PPARγ. Currently, the reason for this discrepancy is not clear. One possible explanation is that the effect of IL-1β on the expression of each PPAR isoform is cell-type- and context-dependent. These observations suggest that PPARβ/δ may also be involved in the regulation of inflammatory processes in mesangial cells. This speculation is supported by our findings that PPARβ/δ was functionally expressed in human glomerular mesangial cells, where its activation and overexpression markedly enhanced basal and IL-1β-induced COX-2 expression and PGE2 production, which may accelerate glomerulonephritis by promoting the biosynthesis of the proinflammatory chemokines and cytokines responsible for the recruitment of leukocytes from circulation to the glomeruli. To date, the role of PPARβ/δ in the inflammatory process remains controversial. Both pro- and anti-inflammatory activities have been reported depending on the cell type and the mechanism of action. Although it is generally believed that PPARβ/δ exerts an anti-inflammatory effect in most extrarenal cell types, based on our findings, it may promote glomerular inflammation by enhancing COX-2-derived PGE2 in human mesangial cells.

SIRT1, a NAD(+)-dependent deacetylase, regulates a broad range of pathogeneses of many chronic diseases, including metabolic and renal diseases [9,52]. SIRT1 can suppress the expression of multiple genes involved in the inflammatory process, including NF-κB, interleukin-6, and MCP-1 [53,54]. It has been previously reported that SIRT1 also contributes to the regulation of COX-2 expression and PGE2 production in many types of cells. SIRT1 activation could increase the COX-2 expression of renal medullary interstitial cells both in vitro and in vivo [37]. In addition, PPARβ/δ activation is capable of inducing SIRT1 expression [55,56]. These findings strongly suggest that SIRT1 might mediate the induction of COX-2 expression and PGE2 production by PPARβ/δ. As expected, we found that PPARβ/δ activation increased the SIRT1 expression, and SIRT1 inhibition or knockdown significantly attenuated the stimulatory effect of PPARβ/δ on the IL-1β-induced COX-2 expression and PGE2 production in human glomerular mesangial cells.

**Figure 8:**
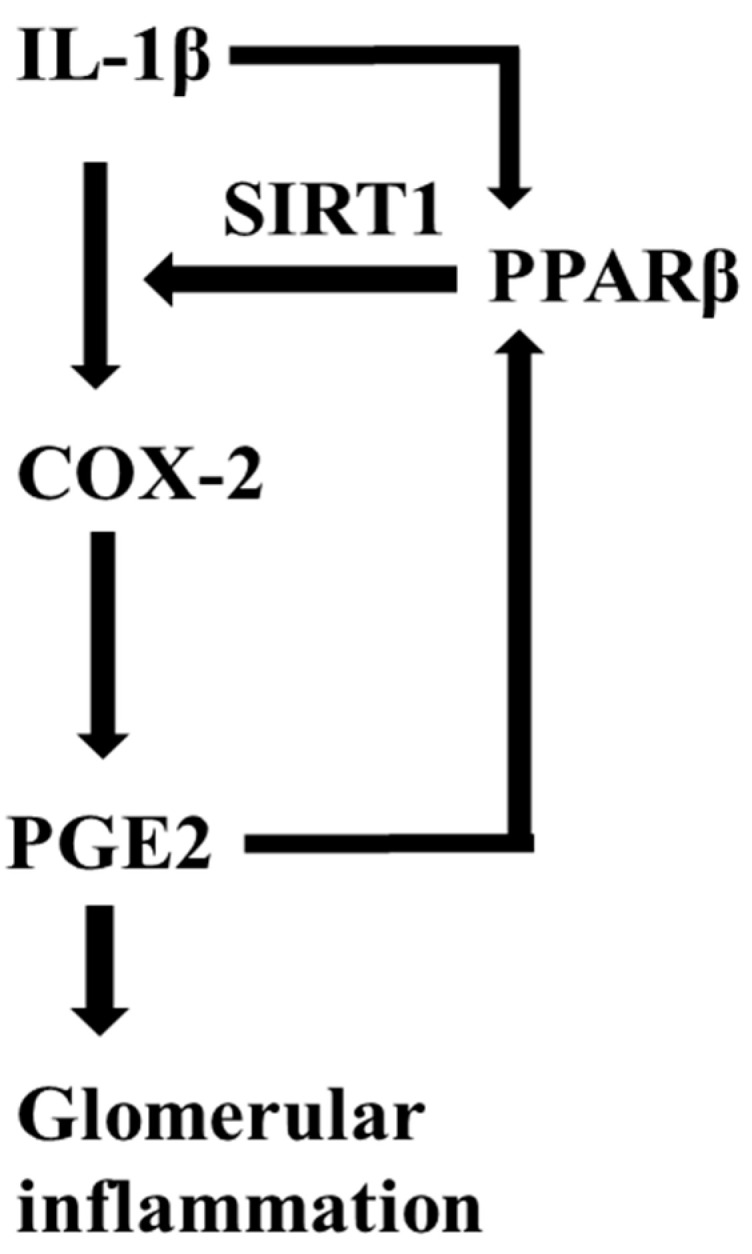
Proposed model of the vicious cycle between PPARβ/δ and COX-2 in IL-1β-induced PGE2 production in human mesangial cells. IL-1β could induce PPARβ/δ expression in both dose- and time-dependent manners accompanied by increased COX-2 expression and PGE2 production. The activation of PPARβ/δ exacerbated the IL-1β-induced COX-2 expression and PGE2 production essentially through inducing SIRT1 expression and activity to promote glomerular inflammation. PPARβ: PPARβ/δ.

In the present study, we provided compelling evidence that the PPARβ/δ-SIRT1-COX2 axis may play an important role in the pathogenesis and progression of glomerulonephritis (Figure 8). The interplay of PPARβ/δ and SIRT1 drove a vicious cycle in promoting the IL-1β-induced COX2 expression and PGE2 production in glomerular mesangial cells. Firstly, we provided clear evidence that PPARβ/δ promoted SIRT1 expression. Secondly, we were able to show that the stimulatory effect of PPARβ/δ on the IL-1β-elicited COX-2 expression was dependent on SIRT1. Thirdly, we found that IL-1β markedly upregulated PPARβ/δ expression. Finally, as previously reported, COX-2-derived PGE2 could indirectly transactivate PPARβ/δ through the PI3K/Akt signaling pathway [57]. Therefore, breaking the PPARβ/δ-SIRT1-COX-2 axis may represent an attractive option for the treatment of glomerulonephritis.

## 4. Materials and Methods

### 4.1. Reagents and Antibodies

All the reagents, including insulin-transferrin-sodium selenite media supplement (ITS), recombinant human IL-1β, GW0742, nicotinamide, and EX527, were purchased from Sigma Chemical Company. Adenoviruses expressing PPARβ/δ (Ad-PPARβ/δ) and TTA (Ad-TTA) were kindly provided by Professor Nanping Wang of the East China Normal University Health Science Center, Shanghai, China. Adenoviruses expressing SIRT1 (Ad-SIRT1), SIRT1 siRNA (Ad-siSIRT1), and the control adenoviruse (Ad-siControl) were generously provided as gifts by Professor Yongsheng Chang of the Beijing Union Medical College, Beijing, China. Antibodies, including β-actin (Santa Cruz sc-47778, Dallas, TX, USA), COX-1 (Cayman Chemical 160109, Ann Arbor, MI, USA), COX-2 (Cayman Chemical 160106), PPARα (Santa Cruz sc-1982), PPARγ (Santa Cruz sc-1981), PPARβ/δ (Abcam ab8937, Cambridge, UK), and SIRT1 (Abcam ab32441) were used in the present study.

### 4.2. Human Mesangial Cell Culture

An established human mesangial cell (hMC) line was used in all the experiments (kindly donated by Dr. Xiongzhong Ruan, UCL Medical School, London, UK). Human MCs were immortalized by transfection with T-SV40 and H-ras oncogenes, retaining many of the morphological and physiological features of normal human MCs. The cells were cultured in RPMI-1640 medium (GIBCO, New York, NY, USA) supplemented with 10% fetal bovine serum (FBS) (GIBCO, New York, NY, USA), 2 mmol/L glutamine, 100 unit/mL penicillin, 100 g/mL streptomycin, 5 g/mL insulin, 5 g/mL human transferrin, and 5 ng/mL sodium selenite at 37 °C in the presence of 5% CO_2_. The cells were split weekly and the media changed twice a week.

### 4.3. Quantitative Reverse Transcription-Polymerase Chain Reaction (RT-PCR)

For the qRT-PCR analysis, the total RNA was isolated from the cells with a commercial mRNA isolation kit (Biotek, Norcross, GA, USA). The isolated total RNA (2 μg) was reverse-transcribed with a RevertAid TM First STRAND cDNA Synthesis Kit (MBI Fermentas, Burlington, ON, Canada), following the manufacturer’s directions. The cDNA was used as a template in the PCR reaction with SYBR Green 1 (Bio-rad, Hercules, CA, USA). The following primers were used: PPARβ/δ_forward: 5′-TGC AGG CTT AGG TCC TCA CT-3′; PPARβ/δ_reverse: 5′-GAG GGA ACC CTG CCT ACT TC-3′; COX-2_forward: 5′-AGA AGG AAA TGG CTG CAG AA-3′; COX-2_reverse: 5′-GCT CGG CTT CCA GTA TTG AG-3′; SIRT1_forward: 5′-CTT CTT GGA GAC TGT GAT GTC-3′; SIRT1_reverse: 5′-GTT CTT CTA AAC TTG GAC TCT G-3′; β-actin_forward: 5′-AGC CAT GTA CGT AGC CAT CC-3′; and β-actin_reverse: 5′-GCT GTG GTG GTG AAG CTG TA-3′. The PCR reactions were carried out at 95 °C for 5 min, followed by 35 cycles of 95 °C for 30 s, 59 °C for 30 s, and 72 °C for 30 s, with a final extension at 72 °C for 5 min. β-actin was used as an internal control. The resulting products were analyzed using 1.5% agarose gel electrophoresis.

### 4.4. Western Blot Analysis

Drug-treated or adenovirus-infected hMCs were firstly rinsed twice with ice-cold PBS and then scraped off the six-well plates and lysed in 100 L ice-cold lysis buffer containing 150 mmol/L NaCl, 20 mmol/L Tris-HCl (pH 7.4), 0.1% SDS, 1 mmol/L EDTA, and 1% Triton X-100. The cell lysate was centrifuged at 12,000 rpm for 5 min at 4 °C after sonication; then, the supernatant was collected. The protein content in the supernatant was quantified with a BCA (bicinchoninic acid) Protein Assay Kit (Vigorous Biotechology Beijing Co., Ltd., Beijing, China). The samples were boiled in SDS sample buffer (313 mmol/L Tris-HCl, pH 6.8, 2% SDS, 50% glycerol, 50 mmol/L dithiothreitol (DTT), and 0.05% bromophenol blue) for ten minutes. An amount of 30 μg total protein was separated using 10% SDS-PAGE, and the protein was electrotransferred onto polyvinylidene difluoride (PVDF) membranes (Immobilon; Millipore, Bedford, MA, USA). The membranes were incubated at room temperature for 1 h in TBS-T (Tris-buffered saline containing 0.1% Tween 20) containing 5% skimmed milk for blocking nonspecific binding sites. The blocked membranes were incubated with a 1:1000 dilution of anti-β-actin or anti-COX-2 polyclonal antibodies or a 1:500 dilution of anti-COX-1, anti-SIRT1, anti-PPARα, anti-PPARβ/δ, or anti-PPARγ antibodies at 4 °C overnight. The membranes were washed for 5 min with TBS-T buffer 3–5 times and then incubated with 1:3000 horseradish peroxide (HRP)-conjugated-anti-rabbit or horseradish peroxide (HRP)-conjugated-anti-mouse secondary antibody at room temperature for one hour. The membranes were washed as described above. Finally, the membranes were developed with ECL. The images were analyzed and quantified with Scion Image software (Scion Corporation, Frederick, MD, USA). 

### 4.5. Transient Transfection and Luciferase Assay

The PPARβ/δ transcriptional activity was determined with a luciferase reporter experiment. The cells were transiently cotransfected with PPRE-luciferase plasmid reporter (PPRE-luc) and β-galactosidase vector using lipofectamine-plus reagent (Gibco-BRL, Rockville, MD, USA) for 24 h, followed by different stimulations as indicated. The stimulated cells were washed twice with ice-cold phosphate-buffered saline (PBS) and then harvested in 100 μL reporter lysis buffer (Promega, Madison, WI, USA). The luciferase activities were measured using a luminometer (Auto LUMIcounter Nu1422ES; Nition, Tokyo, Japan).

### 4.6. Adenovirus Infection of hMCS

Subconfluent hMCs were infected with Ad-GFP, Ad-SIRT1, Ad-siControl, or Ad-siSIRT1, separately. The same doses of Ad-GFP and Ad-siControl were used as negative controls in our studies. Particularly, Ad-PPARβ/δ and Ad-TTA without 0.1 g/mL tetracycline were needed to overexpress PPARβ/δ, whereas Ad-PPARβ/δ and Ad-TTA with 0.1 g/mL tetracycline were used as controls. The infected hMCs were cultured for 24 h before the experimental assays. The hMCs were also infected with Ad-siSIRT1 for 24 h following PPARβ/δ activation.

### 4.7. Measurement of Prostaglandin E2 Levels

The medium of the cultured human mesangial cells in a six-well plate was collected, centrifuged, and stored at −80 °C until PGE2 measurement. The PGE2 content in the medium was measured with a PGE2 High-Sensitivity Immunoassay kit (Cayman Chemical, Ann Arbor, MI, USA). The protocol for measuring the PGE2 concentration was described in the manufacturer’s directions provided with the kit. The concentration of PGE2 in each cultured well was normalized to the protein content of the cells in the corresponding well. 

### 4.8. Immunofluorescence Staining

The hMCs were fixed in 4% paraformaldehyde in phosphate-buffered saline (PBS) for 10 min, followed by three washes with PBS. The fixed cells were permeabilized with 0.5% Triton X-100 in PBS for 10 min at room temperature and washed three times with PBS. The cells were then blocked with 1% bovine serum albumin (BSA) in PBS at room temperature for 10 min. To detect the PPARβ/δ protein expression, a rabbit anti-PPARβ/δ polyclonal antibody (ab8937, 1:100 dilution in 0.5% BSA) was incubated with the cells overnight at 4 °C. After washing three times with PBS, the cells were incubated with appropriate Dylight 594 (1:100) (red)-conjugated secondary antibodies (Jackson ImmunoResearch, West Grove, PA, USA) for 1 h at room temperature, followed by three washes with PBS. The nuclei were stained with DAPI (1:3000) for 5 min at room temperature. The stained cells were investigated using an Olympus BX-60 fluorescence microscope (Olympus, Tokyo, Japan).

### 4.9. Statistical Analysis

The data were given as means ± SD. The significance of difference was determined with an unpaired, two-tailed Student’s *t*-test or ANOVA. *p* < 0.05 was considered to be statistically significant.

## 5. Conclusions

Our study demonstrated that PPARβ/δ is functionally expressed in human glomerular mesangial cells. IL-1β could induce PPARβ/δ expression in dose- and time-dependent manners accompanied by increased COX-2 expression and PGE2 production. The activation of PPARβ/δ exacerbated the IL-1β-induced COX-2 expression and PGE2 production, essentially, through inducing SIRT1 expression and activity. Our findings suggested a vicious cycle between PPARβ/δ and COX-2 in promoting proinflammatory PGE2 production, which may serve as a target for the treatment of glomerulonephritis.

## Data Availability

The data presented in this study are available in the article.
